# 3D Printing of Dental Prostheses: Current and Emerging Applications

**DOI:** 10.3390/jcs7020080

**Published:** 2023-02-15

**Authors:** Fereshte Rezaie, Masoud Farshbaf, Mohammad Dahri, Moein Masjedi, Reza Maleki, Fatemeh Amini, Jonathan Wirth, Keyvan Moharamzadeh, Franz E. Weber, Lobat Tayebi

**Affiliations:** 1 Department of Endodontic, Faculty of Dentistry, Tabriz University of Medical Sciences, Tabriz P.O. Box 5163639888, Iran; 2 Department of Medical Nanotechnology, Faculty of Advanced Medical Sciences, Tabriz University of Medical Sciences, Tabriz P.O. Box 5163639888, Iran; 3 Research Center for Pharmaceutical Nanotechnology, Biomedicine Institute, Tabriz University of Medical Sciences, Tabriz P.O. Box 5163639888, Iran; 4 Department of Pharmaceutics, School of Pharmacy, Shiraz University of Medical Sciences, Shiraz P.O. Box 6468571468, Iran; 5 Department of Chemical Technologies, Iranian Research Organization for Science and Technology (IROST), Tehran P.O. Box 33535111, Iran; 6 School of Dentistry, Shahed University of Medical Sciences, Tehran P.O. Box 5163639888, Iran; 7 School of Dentistry, Marquette University, Milwaukee, WI 53233, USA; 8 Hamdan Bin Mohammed College of Dental Medicine (HBMCDM), Mohammed Bin Rashid University of Medicine and Health Sciences (MBRU), Dubai P.O. Box 505055, United Arab Emirates; 9 Center for Dental Medicine/Cranio-Maxillofacial and Oral Surgery, Oral Biotechnology and Bioengineering, University of Zurich, Plattenstrasse 11, CH-8032 Zurich, Switzerland

**Keywords:** 3D printing, dental prostheses, dental implants, dental crown and bridge

## Abstract

Revolutionary fabrication technologies such as three-dimensional (3D) printing to develop dental structures are expected to replace traditional methods due to their ability to establish constructs with the required mechanical properties and detailed structures. Three-dimensional printing, as an additive manufacturing approach, has the potential to rapidly fabricate complex dental prostheses by employing a bottom-up strategy in a layer-by-layer fashion. This new technology allows dentists to extend their degree of freedom in selecting, creating, and performing the required treatments. Three-dimensional printing has been narrowly employed in the fabrication of various kinds of prostheses and implants. There is still an on-demand production procedure that offers a reasonable method with superior efficiency to engineer multifaceted dental constructs. This review article aims to cover the most recent applications of 3D printing techniques in the manufacturing of dental prosthetics. More specifically, after describing various 3D printing techniques and their advantages/disadvantages, the applications of 3D printing in dental prostheses are elaborated in various examples in the literature. Different 3D printing techniques have the capability to use different materials, including thermoplastic polymers, ceramics, and metals with distinctive suitability for dental applications, which are discussed in this article. The relevant limitations and challenges that currently limit the efficacy of 3D printing in this field are also reviewed. This review article has employed five major scientific databases, including Google Scholar, PubMed, ScienceDirect, Web of Science, and Scopus, with appropriate keywords to find the most relevant literature in the subject of dental prostheses 3D printing.

## 1. Introduction

Shifting from a traditional and manual workflow to a digital one is one of the major tasks of the dental community in today’s world. The integration of new technologies, techniques, and instruments, which can be known as a routine practice, is the backbone of such shifting. In the last decade, the field of restorative dentistry has been significantly impacted by the emergence of novel, fully automated, and rapid prototyping techniques to design and fabricate dental prostheses in a three-dimensional (3D) manner [1]. Digital dentistry has witnessed enormous progress, especially with regard to computer-aided design (CAD)/computer-aided manufacturing (CAM) imaging and milling systems, which addressed many challenges in clinical dentistry [2]. Three-dimensional printing is an additive manufacturing procedure included in the most recent wave of technological progress [3]. Nowadays, a wide range of dental treatment trials, including orthodontics, dental implants, mandibular reconstructions, prosthodontic rehabilitation, surgical, and nonsurgical endodontics, have extensively exploited 3D printing technology [4].

This technique utilizes CAD to produce complex 3D constructs with desired geometries, allowing for a highly efficient, low-cost, and patient-specific design approach with the potential for rapid prototyping. Indeed, the advent of novel 3D printing methods for dental application and the availability of related products in the market has attracted attention to this technology, due to its utility and burgeoning research [5,6]. Benefiting from 3D printing, clinicians may repair and replace damaged dental structures using specific biomaterials [7].

Unlike the 3D printing of complex and detailed structures, such as dental casts [8,9], orthodontics [10,11], and surgical guides [12], the process of fabricating single-unit crowns is relatively simple, allowing clinicians to perform same-day fabrication. It only takes a few minutes to scan a 3D model and send it to a chair-side 3D printer to print a unit crown. The clinician can then easily remove the supports and instantly cement the printed prosthesis. This new procedure can enhance clinical productivity and efficiency by providing an alternative to the analog methods of fabricating provisional restorations. Despite the availability of this technology, it is still limited by issues with material compatibility, availability, cost-efficiency, and operator calibration [13]. Despite the existence of the technology that allows clinicians to carry out these procedures, there are still some bottlenecks, such as a lack of experience in applying the appropriate dental 3D printers and materials, in relation to compatibility, availability, and cost-efficiency, which should be addressed. In recent studies, dental prostheses have been obtained with precise 3D printing systems using a variety of materials, including zirconia [14], pure titanium [15], and polymer-based composites [16].

In general, 3D printing is becoming increasingly popular in dentistry because it allows practitioners to create highly customized and precise dental prosthetics, such as crowns, bridges, and implants. This can be particularly beneficial for patients who have unique dental anatomy or who require customized solutions, due to injury or other conditions.

Using 3D printing technology, dentists can create models of a patient’s teeth and jaw that can be used to plan and prepare for various dental procedures. They can also create physical models of the finished product, which can be used to ensure a proper fit and make any necessary adjustments before the final product is produced.

In addition to improving patient care, 3D printing can also be more efficient and cost-effective than traditional manufacturing techniques, as it allows for the production of complex and customized products on demand, without the need for large quantities of inventory or specialized equipment.

Although 3D printing is known as a storm in the world of technology and manufacturing for different fields, it is still at the beginning of its growth in dentistry. The importance of the present study is providing a review of the recent progress regarding the use of 3D printing in dentistry and, more specifically, in dental prostheses, which can include the crowns, bridges, caps, dentures, or surgical tools. Employing different groups of materials that can fundamentally be handled by different methods of 3D printing has special significance in dental applications, which is discussed in this manuscript. There are various limitations and challenges regarding the use of 3D printing for the manufacturing of dental prostheses, which are elaborated in this article.

Five key scientific databases, including Google Scholar, PubMed, ScienceDirect, Web of Science, and Scopus, were employed in order to search the articles with the most relevant subject and scope to the title of the manuscript. The keywords used for the search include 3D printing AND dental prostheses OR dental crown OR dental bridges OR dental implants. This review covers the articles published from 1999 to 2022.

## 2. General Overview of 3D Printing Techniques, Advantages, and Disadvantages

Herein, the results of the review works have been mentioned by details. The details of 3D printing technics and their pros and cons are documented. Investigators developed the first 3D printer at the Massachusetts Institute of Technology (MIT) in 1993 by expanding the techniques of 2D inkjet printing [17]. A few other commonly used printing methods have been developed since then. Nowadays, with recent advances in inventing 3D printing machines with different mechanisms, from extrusion to sintering, there are various 3D printers, both in dentistry and industry. Among the 3D printing techniques, stereolithography (SLA/SLG), fused deposition modeling (FDM), selective laser sintering (SLS), selective laser melting (SLM), powder binder printers (PBP), and digital light processing (DLP) are the most commonly used techniques [5,18].

1. Briefly, the extrusion-based methods employ a nozzle with a particular diameter to dispense the desired material in three axes, controlled by a computer. These methods rely on a continuous ejection of extruded material driven out of the nozzle, mechanically or pneumatically, to form a 3D structure at the centimeter scale [19]. In the FDM method, which is an extrusion-based technique, different materials, such as thermoplastic polymers, are melted and then driven out from the nozzle. The melting material deposition on the instrument support is concomitant with the cooling down of melted material, which is the main step for fabricating favorable 3D structures [20]. Extrusion-based techniques with low costs can quickly construct basic and less complex models. Recently, the use of microfluidics to develop the FDM method has been progressed. This gives them advantages, such as affordability and low cost. The application of microfluidic in the fabricating of dental prostheses development has attracted much attention [21].
2. In the laser melting/sintering method, high-power pulsed laser light increases the temperature of specific areas to weld or sinter the added material on a three-axis moving stage. Using the SLS technique, a wide range of thermoplastic materials, including thermoplastic polymers, glass, ceramics, and metals, can be fused. Then, new surface layers can be created by refreshing the surfaces with a roller or blade. Finally, a powder form of material is applied to each sintered layer. One of the most important advantages of sintering techniques is that they lead to an autoclavable product that can be handled safely through common dental treatments [22]. The prostheses are easily duplicated at the dentistry office by applying computer-aided software and a desktop SLA 3D printer. This increases the rate and quality of manufacturing at a low moment [23].
3. In the digital light processing (DLP) method, a projector light source cures the liquid resin layer-by-layer, and each layer is created upside down. To overcome problems during the DLP method, such as shrinkage or increasing the error when the size of the point out varies, the combination of DLP and FDM models has been proposed. The FDM accuracy for full-arched dental models and FDM inaccuracy for crown prostheses have been considered. So, a hybrid method of DLP (for special die) and FDM (for full dental model) is suggested [24].
4. In the powder binder printers (PBP) method, the apparatus infiltrates pigmented liquid droplets layer-by-layer using an inkjet head [5]. However, utilizing biocompatible powders in tissue engineering is important. Calcium phosphate-based materials, as a reactive component, will be a good material (because of their similarity to dental sources) for implant applications [25].
5. Lastly, lithography-based techniques employ photopolymers as the printing material. These photopolymers are directly exposed to the laser or a UV light beam or via the lithography-based ceramic manufacturing technique, while the stage moves in different directions to obtain the 3D structure. In these techniques, motorizing mirrors help concentrate the light beam on the surfaces containing the photoreactive liquid resin meant to be fused. Then, the curved surface is recoated by a wiper. This process is followed by another fusion step to infiltrate or stain the particular areas of the printed material [22]. These photopolymers have various properties, such as elemental composition microstructure and fracture mechanics. Ucar et al. compared these features in three products. The lithography ceramic-based technique was the most promising [26].

**Figure 1.**
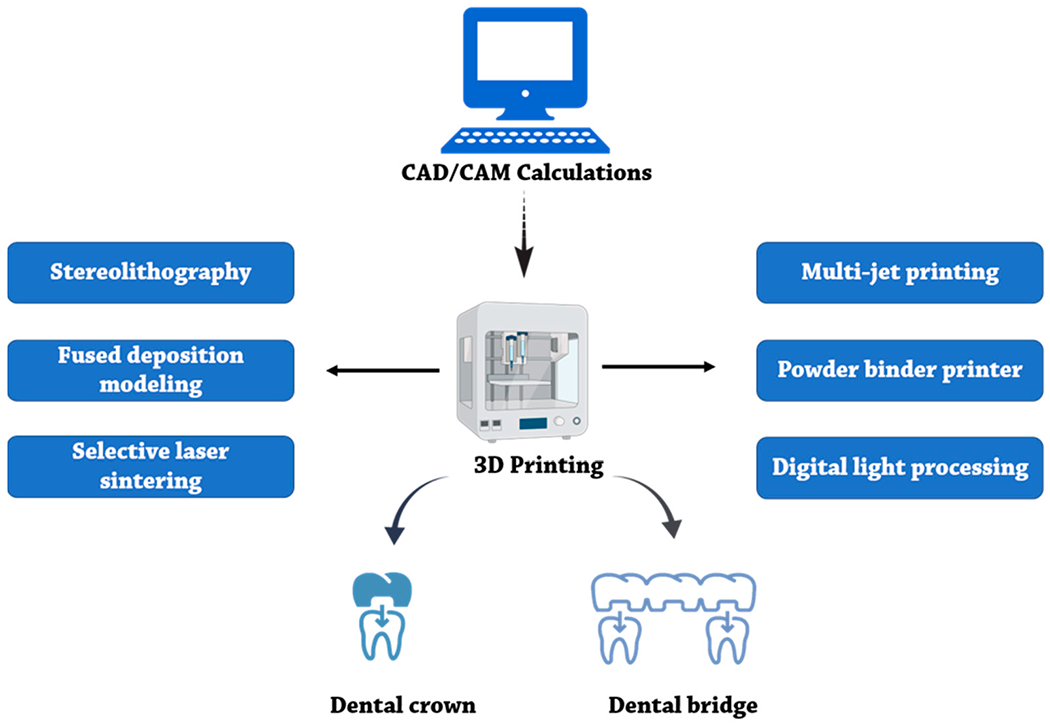
Different 3D-printing models for fabricating dental crowns and bridges.

The pros and cons of the most commonly used 3D printing techniques are presented in Table 1. Additionally, the various methods are mentions at Figure 1.

### 2.1. Three-Dimensional Printing and Modeling

Three-dimensional printing has the ability to be concomitantly exploited with other computer-aided technologies, such as core-beam computed tomography (CBCT) and computer-aided design (CAD)/manufacturing (CAM) [4]. Digital oral scanners make it possible to perform a reliable 3D modeling of teeth by using a computer [32]. Additionally, fixed prostheses can be fabricated by these scanners without any need for conventional working models [33].

Today, numerous kinds of fabricating methods have been developed that are capable of shaping the most typical materials. Different types of 3D printing technologies, including stereolithography apparatus, SLS, FFF [34,35], digital light processing, and MJP, can be used to perform 3D modeling.

According to ISO 5725-1:1994/Cor 1:1998, an accurate 3D printing method has trueness and precision concomitantly. In a recent study, using three different technologies, including SLA, DLP, and MJP, they were able to design and manufacture three models of dental prostheses with various resins. Analysis showed that MJP presented meaningfully higher accuracy and trueness than DLP and SLA techniques if the presence of 3D color map analysis for MJP shows surface roughness at the lowest level [36].

Two methods for producing working dental models are obtained by CAD/CAM modeling: milling and 3D printing. Using patient data from oral scanning is the input of the dental modeling. (1) The milling method has more disadvantages (e.g., unnecessary missing over milling, high cost, and time loss over the fabricating process). (2) On the other hand, the 3D printing method has more advantages (e.g., fabricating favorite prostheses, prostheses models with a minimum amount of materials, and the capability to create multiple products at a time) [37]. CAD/CAM has different software choices (e.g., Slic3R and Geomagic, respectively). The most important step in manufacturing the prototype is the progress of a suitable CAD solid model. Newly, hybrid manufacturing (HM) combines various fabricating processes methods of additive and subtractive technics and has important attention [38]. Additionally, using the CAD/CAM methods will promote the yield of prostheses manufacturing [39]. These techniques are employed widely in the fabrication of dental crowns and bridges, with an effective role in clinical settings [40]. For example, Joo et al. reported a clinical case that described a complete ceramic crown and interim fabrication for a 44-year-old man using CAD/CAM modeling and 3D printing techniques [41].

One CAD/CAM modeling capability is determining prostheses’ marginal and internal fits, such as crowns or bridges. A recent study evaluated the marginal and internal fit of feldspathic ceramic crowns by utilizing a tomography analysis [42]. They measured 2D marginal-internal fit and 3D volumetric fit and compared them between crown and reference dies. They found that the mean marginal fit between three different methods was 113.2 μm. These scanning methods will help clinicians to fabricate better prostheses. In another study, Abdullah et al. compared the CAD/CAM technique vs. conventional methods. They investigated the effect of CAD/CAM techniques on the marginal and internal fit. They evaluated a marginal gap in provisional crowns. These were fabricated by utilizing four kinds of resin by applying low-viscosity silicone impression material. They informed a mean marginal gap of 47–193 μm [43]. Additionally, Yao et al. documented a marginal cleft of 150–280 μm. They fabricated provisional crowns utilizing four different resin types [44].

**Figure 2.**
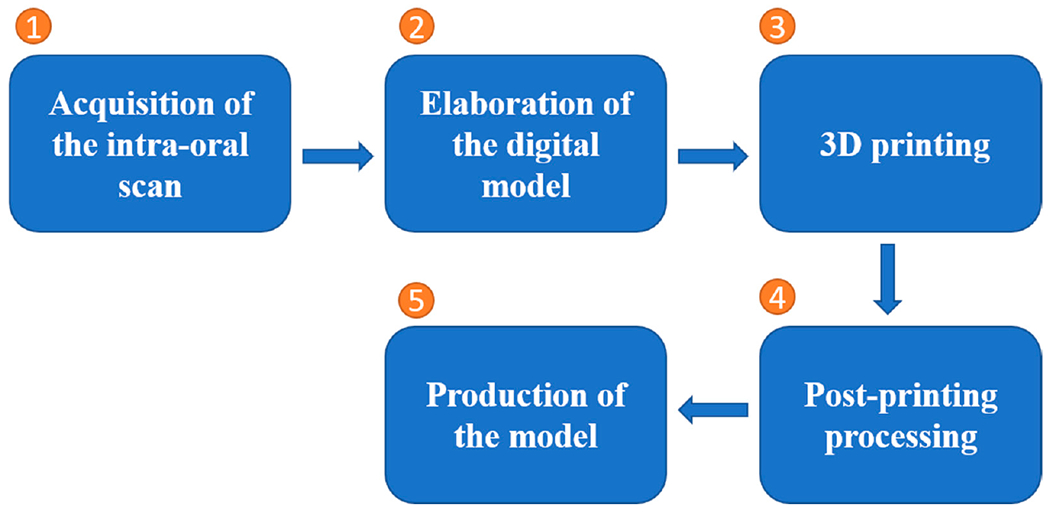
Five steps of clinical 3D printing workflow in dentistry.

The clinical digital workflow can be divided into five parts, which are shown in Figure 2 [45].

There are several software programs that are commonly used for the fabrication of dental prosthetics, surgical guides, and aligners [46].

For prosthetics, CAD/CAM (computer-aided design/computer-aided manufacturing) software is often used to design and mill crowns, bridges, and other dental restorations. Examples of CAD/CAM software used in dentistry include CEREC, 3Shape, and Dental Wings [47–49].

For surgical guides, specialized software is used to plan and fabricate guides that help surgeons accurately place dental implants. Examples of surgical guide software include Simplant, NobelGuide, and X-Guide [50–53].

For aligners, software is used to create treatment plans and generate 3D models of the teeth that can be used to fabricate the aligners. Examples of aligner software include Invisalign, ClearCorrect, and OrthoPlan [54,55].

### 2.2. Three-Dimensional Materials Characteristics

In prosthodontics, common clinical procedures, including the fabrication of crowns and bridges, have gained attention to be performed by additive manufacturing techniques. The reliability and precision of traditionally fabricated dental restorations can be affected by human errors, due to their labor intensiveness [56]. Regarding the marginal gap, occlusal fit, and internal discrepancies of dental restorations, it has been found that both milling and additive manufacturing techniques are more accurate and precise, compared to manual techniques [57]. Similar results were also obtained by other scientists regarding marginal fit [58]. In addition to these results, a systematic review performed by Papadiochou et al. has shown that the identity and quality of restorative 3D printable materials can affect the performance of CAD/CAM processes [59]. In other research, Yoo-Geum Jeong et al. used the root mean square (RMS) value in their study, and according to the results, less value of the RMS in 3D-printed models showed that they were more accurate than those manufactured by the milling method [37].

If the provisional restoration is utilized for a long time, visual concerns of patients will be amplified, particularly in anterior restorations. Clinicians should carefully consider the optical properties, as well as their stability over time [60].

Revilla-León et al. evaluated the color of the 3D printable materials and then compared it with conventional acrylic resin-based interim materials [34]. They comprised five various 3D printable dental materials; however, they did not measure the translucency and color stability. Moreover, Shin et al. measured three CAD/CAM blocks and two 3D printable materials′ color stability [61]. Thus, studies on the color and translucency of 3D printable dental materials, as well as their stability over an extended period, are needed. According to CIELAB (International Commission on Illumination) color space, color is used to obtain these visual criteria. It is concluded based on the difference of colors at different times during one hour [62].

The threshold values for clinically apprehensible color and translucency differences are defined as ΔE ≥ 3.7 and |ΔTP| ≥ 2.0, respectively [4,63]. In a recent study, five substances of different resin types were evaluated [4]. These results showed that the color and translucent dental materials for crown and bridge restorations changed over time. These products are fabricated by the DLP technique. One of the most important limitations related to 3D printable dental materials is that the color of materials that are used for 3D printing can be changed over time [4]. Moreover, the colors of 3D printable materials are basically different. In contrast to the color, the translucency of the material undergoes a relatively minor change. Accordingly, the materials used in the 3D printing of dental materials become opaque, darker, and more yellowish during the 6-month storage in the aqueous medium [4].

Karatas et al. [64] investigated the effects of staining on the resin surface properties. More specifically, they studied the effect of bleaching and staining on the surface microhardness, roughness, and color changes (ΔE). After bleaching, a significant decrease of microhardness was observed in the microhybrid composite specimens.

Surface roughness is also one of the important factors used to evaluate the performance of dental materials [65]. In a study, Sobbah et al. [66] were able to study the effect of different layers and thicknesses on surface roughness. The results showed that changing layer thickness and storage time does not affect surface roughness. On the other hand, in their study, Arnold et al. found that roughness parameter and printing angles are the sources of variations in roughness [67].

Jain et al. fabricated 3D-printed resins to evaluate the color stability of dental prostheses. They found that, after diving into denture cleanser solutions, all of the denture base resins had a substantial variation in color [68].

In addition, some novel methods have been carried out to assess the property changes. For example, Warnecki et al. [69] utilized fractal dimension analysis, texture analysis, and wetting angle assessment to evaluate the mechanical characterizes and behavior of the appliances.

Three-dimensional printing constructs have been influenced by various factors during preparation, printing, and post-printing procedures, with direct effects on the mechanical behavior and quality of fabricated components [70]. For example, raster orientation and printing speed are two key printing parameters that impact the specimens feature [70,71].

## 3. Application of 3D Printing in Dental Prostheses

Three-dimensional printing in dentistry, mainly when applied to the fabrication of dental provisional restorations (bridges and crowns), is new, with related studies dating as recently as 2013. Most of these studies focused on the fit of 3D-printed restorations and compared their characteristics with those fabricated by conventional methods (Table 2). Marginal and internal fit is identified factors that have a critical impression on the long-term achievement of dental restorations [72]. The marginal fit of restorations is highly related to their manufacturing technique [73]. Some parameters can influence the marginal fit of prostheses manufactured by 3D printing, such as type of resin, type of printing machine, device calibration, in terms of environmental temperature and moisture, and restoration shape complexity [74]. Table 2 shows the methods and materials used for 3D printing applications in dental prostheses and crowns [75].

**Figure 3.**
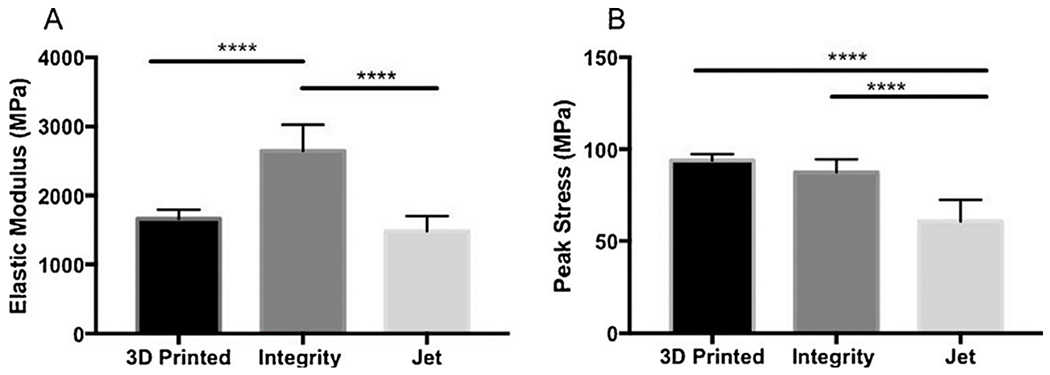
(**A**) Elastic modulus and (**B**) peak stress for 3D-printed specimens versus two conventionally cured provisional materials (Integrity^®^ and Jet specimens^®^). As shown in the figure, no direct correlation between printing layer thickness and peak stress, nor elastic modulus, was documented. The figure is adapted from ref. [77], with permission from Elsevier. ****: statistically significant with *p* < 0.0001.

In a recent study, Lee et al. investigated the internal fit of dental crowns obtained by 3D printing and the CAD/CAM milling method using stainless steel and vinyl-polysiloxane [76]. As a result, the mean discrepancy values were measured to be 141.1 and 91.1 μm for the crowns fabricated by two brands of 3D printing systems and 171.6 μm for those fabricated by the milling system. Furthermore, the internal and marginal fit of the fabricated crowns obtained from 3D printing systems were significantly improved, compared to those fabricated by CAD/CAM milling system. Tahayeri et al. optimized the 3D printing of dental materials for bridge restorations and provisional crowns using a low-cost stereolithography 3D printer and compared their mechanical properties to conventionally cured provisional dental materials [77]. Fourier-transform infrared spectroscopy (FT-IR) analysis and three-point bending methods were employed to evaluate the degree of conversion of the resin and the peak stress and the elastic modulus of 3D-printed bars, respectively. They also compared the obtained results with two conventionally cured provisional materials, Jet^®^, Lang Dental Inc., Wheeling, IL, USA, and Integrity^®^, Dentsply, Charlotte, NC, USA. As reported, there was no direct correlation between the printing layer thickness and peak stress or elastic modulus. The 3D-printed models showed similar and significantly higher peak stress compared to Integrity^®^ and Jet^®^, respectively (Figure 3). Furthermore, compared to Jet^®^ and Integrity^®^, the 3D-printed samples had comparable and significantly lower elastic modulus. Interestingly, the 3D-printed samples also showed an enhanced degree of conversion than those of Jet^®^ and Integrity^®^.

**Figure 4.**
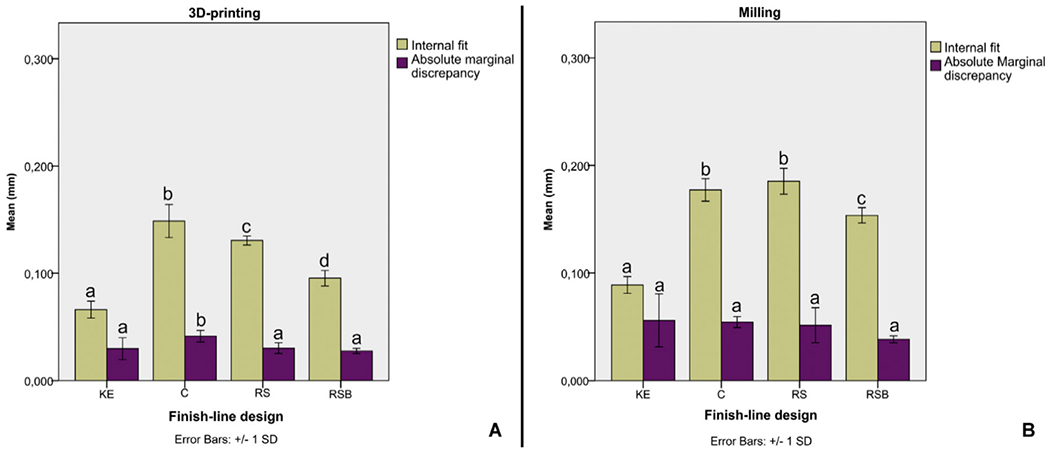
Influence of finish line design on internal fit and AMD in (**A**) 3D printing and (**B**) in milling. Different letters indicate statistical significance between different finish line designs. The figure is adapted from ref. [58], with permission from Elsevier.

Similarly, Alharbi et al. compared the effects of 3D printing and milling methods and different finish line designs on the marginal and internal fit of interim restorations [58]. As reported, the mean internal gaps for milled restorations were 89, 177, 185, and 154 μm, fabricated on a knife-edge (KE), chamfer (C), rounded shoulder (RS), and rounded shoulder with bevel (RSB) finish line designs, respectively. On the other hand, these values for 3D-printed restorations were reported to be 66, 149, 130, and 95 μm, respectively, for KE, C, RS, and RSB, indicating the significantly lower internal gap of 3D-printed restorations, compared to those obtained from milling methods (Figure 4). Furthermore, the 3D-printed restorations showed lower mean absolute marginal discrepancy (30, 41, 30, 28 μm) than the milled restorations (56, 54, 52, 38 μm) for KE, C, RS, and RSB, respectively. The aforementioned results designate that the finish-line design techniques have a lower impact on the fit than the fabrication methods [58].

In another study, Chaturvedi et al. evaluated the marginal and internal fit, using finish line chamfer, rounded shoulder, and rounded shoulder with bevel, of the provisional crowns obtained by three-dimensional (3D) printing, compression molding, and milling methods [81]. The result of that fabrication method and finish line design has a substantial effect on the internal and marginal gap. The minimal marginal gap and the best internal fit in all various finish lines belonged to the 3D printing methods, in comparison with compression molding and milling methods [27].

Despite providing a rapid technique for the fabrication of zirconia dental restorations, CAD/CAM milling systems present a few disadvantages, such as material waste, processing defects, such as microscopic fractures, and inadequate accuracy [98]. Ebert et al. employed direct inkjet 3D printing to fabricate all-ceramic dental restorations using zirconia-based ceramic suspension as the source material [85]. The obtained 3D-printed dental crowns possessed high mean fracture toughness (K_Ic_ = 6.7 MPam^0.5^) and characteristic strength of the ground bars (σ_0_ = 763 MPa) with a 90% confidence interval of [678;859]. Revealed by scanning electron microscopy (SEM), homogeneous cross-sections were apparent, with no significant defects (except for a few process-related ones) on the surface of the specimens. In view of cost-efficiency, this promising method consumes a minimum number of materials.

To further evidence the superior efficiency of 3D printing procedures for the manufacturing of dental prostheses compared to conventional techniques, Eftekhar Ashtiani et al. employed intraoral scanning and 3D printing of the pattern for the fabrication of intracoronal restorations and compared their dimensional accuracy to those obtained from the conventional fabrication of a resin pattern [79]. Interestingly, the conventional method resulted in more accuracy than the 3D printing, regarding impression making and the fabrication of intracoronal restorations. However, the fabricated restorations with both methods yielded a clinically acceptable fit.

Similarly, Homsy et al. compared the internal and marginal fit accuracy of lithium disilicate-based inlays manufactured with conventional milling and 3D printing methods [80]. Here, the marginal and internal fit accuracy of the inlays fabricated by digital impressions and subtractive milling of wax patterns were significantly better than those of the conventional impression/fabrication and 3D printing techniques. It is worth noting that the fit values measured for 3D-printed wax patterns were similar to those of the conventionally waxed inlays.

Mai et al. examined the fit of interim crowns (proximal, marginal, internal axial, and internal occlusal regions) made by photopolymer jetting 3D printing and compared it to compression molding and milling techniques [57]. According to this study, in the marginal and proximal regions, the milling and 3D printing methods exhibited more accurate results than the molding method. Furthermore, 3D printing resulted in the most accurate values in the occlusal region. The internal discrepancies were the highest for the milling method, meaning that the fit of temporary crowns, particularly in the occlusal region, can be efficiently enhanced by taking advantage of polymer-jet 3D printing.

Fathi et al. reported similar results, stating that the 3D-printed wax crowns were more accurate, in terms of internal and marginal fit, than milled and manually fabricated wax crowns [82]. Furthermore, conventional hand carving and milling of wax crowns both resulted in significant discrepancies in occlusal gap points, when compared to additive methods, demonstrating 3D designing and manufacturing of wax patterns for complete crowns as a more accurate procedure [99].

As the fracture load is a critical characteristic for dental restorations, it is crucial to assess this index for structures that novel approaches, such 3D printing, have manufactured [100]. To this end, Zimmermann et al. determined and compared the fracture load of crowns made of three particle-filled composite CAD/CAM materials, including Cerasmart (GC Corporation), Brilliant Crios (Coltène AG; Altstätten), and Lava Ultimate (3M ESPE), and one 3D-printed composite (els-3D Harz; Saremco Dental AG) as a function of three different material thicknesses (0.5, 1, and 1.5 mm) [83]. Amongst all groups, Brilliant Crios and els-3D Harz, respectively, showed the highest mean loading forces before fracture, which were measured to be 1580.4 N and 1478.7 N, with 1.5 mm thickness. The fact is that the fracture loading force mainly depends on the respective material and thickness; therefore, none of the 0.5 mm ceramic crowns (as group control) survived the fatigue testing, while all the resin-based crowns did. Consequently, regardless of the fabrication mode (CAD/CAM or 3D printing), the particle-filled composite resins may play an essential role in manufacturing minimally invasive restorations with good mechanical properties [83]. A wide range of complications can be developed as consequences of the marginal discrepancy between the abutment and the restoration material [101].

Similar studies on the fabrication of CAD/CAM-based temporary crowns and evaluation of the internal matching were conducted by Lee et al. First, they scanned the readymade stainless model using digital scanners and designed it with software CAD/CAM. Then, they used 3D printers to fabricate dental crowns. Zirkonzahn (3D milling system), Stratasys, and Dentis (3D printing system) technology were used to fabricate the crowns [76]. Vipi block, VeroGlaze MED620, and ZMD-1000B resin were applied in the fabrication. For each group, ten files were made. The mean ± standard deviation (SD) values of marginal discrepancy were found to be 171.6 ± 97.4, 149.1 ± 65.9, and 91.1 ± 36.4 for the CAD/CAM milling group, Stratasys group, and Dentis group, respectively. *p*-values for the 3D printing groups (consisting of Stratasys and Dentis groups) were lower, compared to that for the CAD/CAM milling group. According to the significant level of the *p*-value (a *p*-value less than 0.05 was considered significant), the mean discrepancy values were found to have statistically significant differences. Accordingly, a high level of completion is an advantage that makes the 3D printing method a suitable method to be applied in the production of dental prostheses, in addition to the interim restoration production [76].

The solidification of liquid polymer resins, including resins, photopolymers, and transparent resins, is a process in additive manufacturing performed by UV light in digital light processing (DLP) printing technology [102]. Applications of DLP in dentistry include the fabrication of dental models, dental implants, cochlear implants, and dental restorations. Moraru et al. used this technique to fabricate dental prostheses [92]. The advantages of this method are its high accuracy in printing different parts, as well as the appropriate surface of the final product [103]. Son et al. fabricated three 3D printing Interim dental crowns by SLA and DLP and milling methods. They focused on the comparison of intaglio surface trueness at each implant. They used CAD/CAM to design and model the crowns, as in previous studies. In the fabricating procedure of interim dental crowns, 3D printing technologies presented higher accuracy than milling [91].

Firlej et al. investigated five materials for 3D printing applications. These resins were UV-curable form. Isopropyl alcohol was sprayed on all of them. This removed the rest of the resins on the implants. They evaluated the effect of artificial aging on the quality of materials in various implants [88]. In some cases, both milling and 3D printing methods are clinically acceptable. For example, H Galeva et al. compared the internal and external accuracy fit of metal-ceramic fixed prosthetic constructions. Additionally, the temperature influence evaluations showed that the differences were not noticeable [93].

Temporary restoration modeling and fabrication are important in various dental applications. Mohajeri et al. investigated the effect of conventional fabricating methods against 3D printing on the marginal fit [94]. By utilizing these three fabrication methods, the restorations were generated via clinically suitable marginal fit.

**Figure 5.**
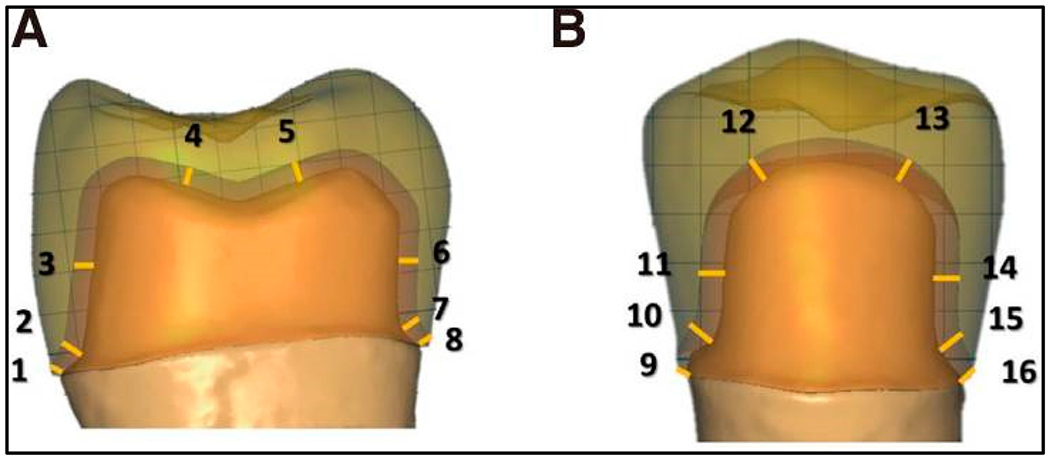
Sixteen measuring points for the marginal and internal gap of the crown. (**A**) Buccopalatal section, (**B**) Mesiodistal section. Marginal gap (MG): 1, 8, 9, 16; cervical gap (CG): 2, 7, 10, 15; axial gap (AG): 3, 6, 11, 14; occlusal gap (OG): 4, 5, 12, 13. The figure is adapted from ref. [95], with permission from Elsevier.

In a recent study, Ryu et al. investigated the effect of different directions, including 120°, 135°, 150°, 180°, 210°, and 225 °C [95]. The best fit was obtained with the shape angles of 180° and 150°. Figure 5 shows the marginal gap from a different view. The orientation of the build is important in the fabrication techniques [104]. For example, Lee et al. examined the accuracy (trueness and precision) of the interim crowns. They utilized a DLP printer and investigated post-curing time. The build angle was 135° [96]. Yu et al. [97] examined the intaglio surface and build angle on the interim crown. Additionally, they determined the effect of build angle, including 0, 120, 135, 150, 180, 210, 225, 240, and 270 degrees, on dental crown fabricates′ mechanical properties. The results showed the build angle effect on the intaglio surface trueness and marginal gap. The recommended angle was between 150 and 210 degrees [97].

A surgical guide in dentistry is a template that is used to accurately position dental implants in the jawbone [105]. Three-dimensional printing can be used to fabricate surgical guides by creating a physical model of the patient′s jaw and teeth based on a digital 3D scan [106,107]. The guide is then created by slicing the digital model and exporting the slice data to a 3D printer [52,108]. Here is a step-by-step overview of the process:

1. Digital 3D scan of the patient′s jaw and teeth: The first step in fabricating a surgical guide using 3D printing is to obtain a digital 3D scan of the patient′s jaw and teeth. This can be achieved using a variety of technologies, including CBCT and intraoral scanners.
2. Design of the surgical guide: Once a digital 3D scan has been obtained, the next step is to design the surgical guide using specialized software. This involves creating a virtual model of the patient′s jaw and teeth and then planning the placement of the dental implants based on the specific needs of the patient.
3. Slicing the digital model: The next step is to slice the digital model of the surgical guide into layers, which can then be exported to the 3D printer. This process involves specifying the thickness of the layers and the type of 3D printing technology to be used.
4. 3D printing: The slice data is then sent to the 3D printer, which creates a physical model of the surgical guide using a variety of materials, such as plastic or metal.
5. Post-processing: Once the surgical guide has been printed, it may need to be post-processed, in order to smooth out any rough edges and ensure that it is accurate and ready for use. This may involve sanding, polishing, and sterilizing the guide.
6. Use in surgery: The surgical guide is then used during the actual implant surgery to accurately position the implants in the jawbone. The guide helps the surgeon to place the implants in the correct location, ensuring that they are properly aligned and positioned for optimal function.

Three-dimensional printing can also be used to fabricate aligners, which are employed in orthodontic treatment to straighten teeth [54,109]. The process begins by creating a 3D model of the patient′s teeth using a digital impression or a physical impression that is scanned into a computer [54]. The 3D model is then used to design the aligners, which are customized to fit the patient′s teeth and apply the necessary forces to move the teeth into their desired positions [110]. The aligners are typically made of a clear, biocompatible plastic material that is suitable for long-term wear in the mouth. Once the aligners have been designed, they can be fabricated using a 3D printer. The finished aligners are then sent to the orthodontist, who gives them to the patient to wear, according to a prescribed treatment plan [54,111].

There are a few limitations to the use of 3D printing for the fabrication of dental surgical guides and aligners in dentistry [52,105,106,108,112,113]:

Material properties: The properties of 3D-printed materials may not be suitable for all dental surgical applications. For example, 3D-printed surgical guides may not be as rigid as those made from other materials, such as stainless steel.

Accuracy: While 3D printing can produce objects with high levels of accuracy, the accuracy of the final printed product may be affected by factors such as the resolution of the printer and the quality of the 3D model used as a reference.

Time: Three-dimensional printing can be a time-consuming process, especially for large or complex objects. This may not be practical in cases where time is of the essence, such as in emergency surgery.

Cost: Three-dimensional printing can be an expensive option, compared to other manufacturing methods, especially for large volume production.

Regulatory considerations: There may be regulatory hurdles to overcome, in order to use 3D-printed dental surgical guides and aligners in clinical practice, as they may be considered medical devices. This may require additional testing and documentation to demonstrate their safety and effectiveness.

There are a variety of clinical case reports regarding the use of 3D printing for dental prosthetics. Three-dimensional printing can be clinically used to create crowns, bridges, and dentures that are custom fit to a patient′s teeth, which can improve the comfort and aesthetic appearance of the restoration [114,115]. For example, Srinivasan et al. performed a double-blind, randomized, and crossover clinical study to show the differences between 3D-printed and milled complete removable dental dentures [115]. They revealed that the cost and time of the workflow are likely same for two groups of patients [115].

One of the important clinical applications of 3D printing in dentistry is the use of this technology for the fabrication of a surgical guide and aligner. Surgical guides created by 3D printing can be used to accurately place dental implants in the jawbone, which can help to reduce the risk of complications and improve the overall success of the surgery. For example, there is a case report by Zoran et al. in which a guide was created by 3D printing technique for implant placement [116]. Its results showed that, considering both surgical and prosthetic aspects, the 3D-printed surgical guide could facilitate having an optimal positioning of the implant [116].

The fabrication of custom dental implants is another clinical application of 3D printing in dentistry. Printing can be used to create custom dental implants that are tailored to the specific shape and size of a patient′s jawbone, which can improve the fit and function of the implant. Par et al. reported a case study on 3D-printed titanium implant [117].

At the clinical stage, 3D printing has various pros and cons [118]. The typical advantages can be attributed to the customizability, flexible and fast design and production, long-term cost, ease of access, minimizing waste, and being environmentally friendly [118,119]. Similar to other fabrication methods, 3D printing has its own drawbacks for clinical applications, including limitations in the material selections, possible post-processing requirements and probable design inaccuracies [118,120].

## 4. Dental 3D Printing Materials

As discussed, there are various 3D printing techniques in dentistry and along with these techniques, a wide range of biomaterials, including hydrogels, ceramics, metals, resins, and thermoplastic polymers, have heavily been explored.

More specifically, there are a number of materials that are commonly used in 3D printing for dental prosthetics, including acrylonitrile butadiene styrene (ABS), polylactic acid (PLA), and a variety of resins. Several synthetic polymers, including poly(ethylene glycol) (PEG) and poly(vinyl alcohol) (PVA), have also been exploited in the 3D printing of dental biomaterials, due to their tailorable mechanical and degradational properties [27,121]. Some newer materials are being developed and used in 3D printing for dental prosthetics. One example of a new material that has been used in the 3D printing of dental prosthetics is bioceramic. Bioceramic materials are ceramic materials that are biocompatible, meaning they are safe to use in the human body [122]. They have been used in a variety of medical applications, including dental prosthetics, due to their strength, durability, and ability to bond with living tissue. Bioceramic materials are also resistant to wear and corrosion, making them a good choice for use in dental prosthetics that will be subjected to high levels of stress and exposure to oral fluids. Some examples of bioceramic materials that have been used in the 3D printing of dental prosthetics include zirconia, alumina, and hydroxyapatite [123,124].

Composite materials that combine ceramics with other materials, such as plastic or metal, are also good options for the 3D printing of dental prosthetics [125]. These materials offer improved strength, durability, and aesthetics, making them suitable for a wide range of dental applications. Additionally, some of the newer 3D printing technologies, such as SLS and electron beam melting (EBM), allow for the use of metal materials, such as titanium and cobalt-chrome, which can be used to create more durable and biocompatible dental prosthetics.

In addition to different fabricating technologies, different materials present other mechanical properties, such as thickness. For example, in a recent study, three resins, including bisacrylic, acrylic, and PMMA, were used for microcomputed tomography of 3D-printed dental crowns. Utilizing them with 3D printing technology presents higher film thickness in dental crowns [126].

### 4.1. Thermoplastic Polymers

Among the various options available for the fabrication of dental 3D printable substances, polymer-based materials are the most commonly utilized materials. Photopolymerization is the most feasible technique for the fabrication of dental resins or polymeric 3D-printed materials [27]. A smoother surface, strong chemical bonding, suitable mechanical strength, and high-quality build resolution can be provided using photopolymerization [6]. Thermoplastic polymers are the most frequently used polymer-based biomaterials for dental 3D printing [127]. The filaments that make the main backbone structural compartments of thermoplastic polymers can flow through the nozzles by applying heat [128,129]. Among these polymers, polyethylene (PE), polypropylene (PP), PLA, and ABS are the most frequently used in the field of 3D printing of polymeric dental biomaterials [130]. Due to the non-toxic properties of PLA against the oral cavity, it is assumed to be more favorable for utilizing in 3D printing, compared to ABS [129,131]. Thermoplastic filament polymers with higher glass transition temperatures, such as polymethyl methacrylate (PMMA) and polyether ether ketone (PEEK), have been recently studied for the fabrication of dental 3D-printed materials [27]. Schönhoff et al. compared the amorphous polyphenylene sulfone (PPSU) with established semi-crystalline polyetheretherketone (PEEK). They found that PPSU can be a suitable material, instead of PEEK. They evaluated the mechanical properties that confirmed recently proposed research [132].

Wieckiewicz et al. [133] investigated the surface roughness, color stability, and elasticity of polyamide-12 (PA) versus polymethyl methacrylate (PMMA) denture-based material as a control. The results suggested that PA showed a higher susceptibility than PMMA to discoloration.

### 4.2. Ceramics

Compared to polymers, ceramics are less frequently used in 3D printing [134]. However, due to their unique properties, ceramics are good candidates to be utilized in stereolithography (SLA) and SLS, in which ceramic powder or a pre-sintered ceramic material are processed to form a structure with strong bonding [1,129,135,136]. A biocompatible microenvironment can be formed in ceramic structure by adding mineral substances, such as hydroxyapatite and β-tricalcium phosphate, which provide calcium and phosphate ions [137]. Moreover, the incorporation of calcium and phosphate mineral phases has been shown to improve cell-to-cell interactions and induce cell differentiation and proliferation, which makes these types of ceramics good candidates for craniofacial applications [138,139]. However, due to the challenges associated with the processing of ceramic powders to high-density structures, the products of the selective laser sintering of these powders are porous structures. Additionally, anisotropic shrinkage and stair-step effects can occur upon additive manufacturing approaches. The above-mentioned challenges have limited the ceramic utilization of ceramics for the development and fabrication of ceramic 3D-printed restorations [129,140,141].

### 4.3. Metals

Metals have been mainly used in selective laser sintering to fabricate dental 3D-printed materials [142]. In dentistry, metallic alloys, including nickel, cobalt-chromium, and titanium alloys have been vastly studied and used [143]. However, due to the possible allergic reactions in the oral cavity, scientists no longer suggest the utilization of nickel alloys in dental metallic materials [144]. Similar to ceramics, the fabrication of metallic biomaterials by selective laser sintering leads to porous products [145]. Due to the negative effect of porosity formation on the strength and resistance of 3D-printed materials, some solutions, such as equipping a vacuum pump with a sintering instrument, have been considered, in order to improve the quality of metallic dental prostheses [7,129,146,147]. Among metallic materials, cobalt-chromium and titanium are the most commonly used alloys for the fabrication of 3D-printed metallic prostheses, due to their strength and ductility [148]. Furthermore, various clinical trials in the field of maxillofacial prostheses have been performed on titanium alloys, specifically Ti_6_Al_4_V [1,129,149].

### 4.4. Others

According to SLM and SLS methods, there are some limitations in utilizing ceramics or metals materials [150]. This matter is because of the thermal shock conditions and high melting temperature in metals and ceramics, respectively [151]. Thus, researchers developed novel approaches to overcome these limitations. For example, in a recent study [152], a combination-producing method, called additive manufacturing, was utilized to make high-density zirconia dental crowns. The authors of this study could fabricate high-density ceramic materials, which are difficult to make with conventional methods.

## 5. Prospects and Conclusions

As a cutting-edge additive technology, 3D printing has played a revolutionary role in most of the scientific fields, to which dentistry is no exception, especially regarding dental prostheses with complex structures [153]. Along with an expanding range of utility for 3D printing technology in dentistry, private office manufacturing with 3D printing seems to be a progressively more realistic prospect [154,155]. More specifically, 3D printing is being employed in dental offices to create a variety of objects, including crowns, bridges, and surgical guides. Three-dimensional printing allows dentists to create customized objects that fit a patient′s mouth perfectly, which can improve the accuracy and effectiveness of dental procedures. It can also help to reduce the turnaround time for certain procedures, as objects can be printed on-demand, rather than having to be ordered and shipped from a lab. Demonstrably, a wide variety of materials can be used to fabricate prostheses with clinically sound results and with similar, and sometimes improved, results, compared to analog and subtractive manufacturing methods [156]. There are a variety of materials that can be used for 3D printing crowns, bridges, surgical guides, aligners, and other dental prosthetics, including ABS, PLA, and various types of resins. The most commonly used material for 3D printing these types of dental prosthetics is a type of resin called a photopolymer. This material is capable of producing highly detailed and accurate objects and is suitable for a wide range of applications. Other materials that may be used for 3D printing of dental prosthetics include stainless steel, titanium, and cobalt-chrome alloys. As research progresses, more materials are being implemented with this technology, providing an expanded repertoire of services. The addition of metal 3D printing is currently being investigated; while more evidence is required, the results could allow for expanding 3D printing services to a convenient in-office framework fabrication for fixed and removable prosthetics [157]. Metal 3D printing involves using a laser to fuse together metal powders into the desired shape. This process allows for the production of highly accurate and complex objects, including dental prosthetics [121]. Some of the advantages of using metal 3D printing for dental prosthetics include the ability to create customized and anatomically accurate objects, the ability to produce objects with complex internal structures, and the ability to produce objects with a high level of detail [158,159]. Additionally, metal 3D printing can be used to produce dental prosthetics that are stronger and more durable than those made with other methods. How this technology will be integrated with commercially available polymer and ceramic-based printers is yet to be seen. However, it is important to note that metal 3D printing can be a more expensive option, compared to other methods of fabrication, and it may not be suitable for all types of dental prosthetics. It is important to note that the choice of material and fabrication method will depend on the specific application, the requirements of the patient, and the clinical indications of the case.

There are several challenges attributed to the 3D printing of dental prostheses [160]. The most important one is accuracy. It is vital for dental prostheses to fit accurately in the mouth, in order to function properly. Three-dimensional printing allows for the creation of highly precise prostheses, but achieving the necessary level of accuracy can be challenging [158,161]. Material selection is another challenge. Dental prostheses need to be made from materials that are biocompatible, durable, and able to withstand the stresses of everyday use. There are a variety of materials that can be used for 3D printing dental prostheses, but choosing the right one for a specific application can be difficult [162,163]. The surface finish is also an important matter to consider in the 3D printing of dental prostheses. They need to have a smooth, polished surface finish, in order to be comfortable in the mouth. Achieving this finish can be challenging when using 3D printing technologies [164,165]. Three-dimensional printing dental prostheses can also be expensive, due to the cost of the equipment and materials [160,166]. This can make it difficult for some patients to afford these types of prostheses. Time can also be an issue in the fabrication of some unique 3D printing dental prostheses. It can be time-consuming, especially when compared to traditional manufacturing techniques [167,168]. This can be a challenge for dental practices that need to produce multiple prostheses in a short amount of time.

In conclusion, 3D printing technology has the potential to revolutionize the way dental prostheses are made. Three-dimensional printing allows for the creation of more comfortable and natural-looking dental prostheses, which can improve the patient′s experience. The use of 3D printing in dentistry has increased in recent years, and it is expected to continue to grow in the coming years by increasing the accuracy, reducing the production time, and lowering the cost. Overall, the prospects for the use of 3D printing in the production of dental prostheses are very promising. It is expected that 3D printing will become increasingly prevalent in dentistry in the coming years, as the technology continues to advance and become more widely adopted.

## Figures and Tables

**Table 1.** Advantages and disadvantages of common 3D printing techniques [27–31].

Technique	Advantages	Disadvantages
FDM or FFF	- Inexpensive - No risk of combustion due to the exploitation of inflammable and non-explosible materials - Applicable for fabrication of intricate structures - Wide range of materials can be subjected to 3D printing by the FDM technique	- Lack of acceptable resolution and accuracy - Additional processing is often needed to smooth or cure the printed surfaces
DLP	- Simple machinery components - Leads to a smooth surface	- Smaller parts and limited areas have greater acceptable resolutions - Surgical guides cannot be printed due to high accuracy considerations
SLA	- Wide range of materials can be printed by the SLS technique - Acceptable accuracy, precision, and resolution - Technique of choice for fabrication of functional prototyping and fine details	- Expensive - Complex post-print processing stages - Use of biohazardous chemicals - Leads to mechanically weak structure - Laser component has an expensive maintenance
MJP	- Create more precise and higher accuracy (trueness) models - Clinically acceptable ranges	- Requirements for dimensional accuracy
SLS	- Inexpensive machinery components - Wide range of materials can be printed by the SLS technique - Suitable for functional prototyping	- The powdered form of starting material must be used - This technique cannot precisely manufacture large parts. - Potential hazards cause high maintenance expenditure - Fine and thin walls (<1 mm) can be printed with difficulties
SLM	- Inexpensive machinery components	- Generate thermal shock condition - High melting temperature

**Table 2.** Summary of recent 3D printing applications in dental prostheses and crowns.

Specimen	Fabrication Method	Resin	Mean Marginal Fit (αm)	Mean Internal Fit (αm)	Mechanical Properties	Ref./Authors/Year
Dental crown	3D printing (Dentis)	ZMD-1000B	91.1	-	-	[76]/Lee et al./2017
Dental crown and bridge	3D printing	Dental crown and bridge		-	Elastic modulus = 1600 MPa (50 μm thickness), peak stress = 100 MPa (25 μm thickness)	[77]/Tahayeri et al./2018
Interim restorations	3D printing (DW028D, DWS)	Temporis^®^ composite	28 (RSB)	66 (KE)	-	[78]/Yang et al./2016
Intracoronal restorations	3D printing (Envision TEC)	WIC 300A envision	10	-	Intracoronal restorations	[79]/Ashtiani et al./2018
Dental inlays	3D printing (ProJet 1200)	Lithium disilicate	39.7	88.8	-	[80]/Homsy et al./2018
Interim restorations	Stereolithography-based 3D printer (DW028D)	hybrid composite resin material (Temporis)	-	-	-	[58]/Alharbi et al./2018
Dental crown	3D printing	-	-	-	-	[81]/Chaturvedi et al./2020
Interim crown	3D polymer jetting (Object Eden 260VS; Stratasys)	VeroGlaze MED620	99	139	-	[57]/Mai et al./2017
Dental crown	3D wax printing (3Z Lab, Solidscape)	Bego Crown Wax	60	115	-	[82]/Fathi et al./2016
Dental crown	3D printing (Freeform Pro 2, ASIGA)	Els-3D Harz	-	-	Mean fracture loading force = 1478.7 N	[83]/Zimmermann et al./2019
Dental interim crown	DLP 3D printing (MoonRay S100)	Nextdent Crown and Bridge Micro Filled Hybrid-MFH	100	-	-	[84]/Çakmak et al./2021
Dental Prostheses	Direct inkjet 3D printing	the ceramic suspension (zirconia powder, TZ-3YS-E)	-	-	-	[85]/Ebert et al./2009
Dental crown	Stereolithographic 3D printing	Resin matrix Bis-GMA/TEGDMA mixture with CQ and 4-EDMAB	-	-	-	[86]/Zhao et al./2021
Dental crown	ASIGA UV MAX	Gr-17.1 temporary	-	-	-	[87,88]/Wesemann et al./2021 and Firlej et al./2021
Dental crown	ASIGA UV MAX	GR-17 temporary	-	-	-	[88,89]/Firlej et al./2021 and Oliver et al./2004
Dental crown	Phrozen Shuffle Lite 3D	NextDent SG Orange	-	-	-	[88,90]/Firlej et al./2021 and Hardiman. et al./2016
Dental crown	Phrozen Shuffle Lite 3D	NextDent C&B MFH	-	-	-	[88]/Firlej et al./2021
Specimen	Fabrication Method	Resin	Mean Marginal Fit (αm)	Mean Internal Fit (αm)	Mechanical Properties	Ref./Authors/Year
Dental Crowns	SLA 3D printing (ZENITH U)	Photopolymer resin ZMD-1000B	-	-	Intaglio surface trueness = 26.7 μm	[91]/Son et al./2021
Dental Crowns	DLP 3D printing (RAYDENT Studio)	Photopolymer resin (RAYDENT C&B)	-	-	Intaglio surface trueness = 27.0 μm	[91]/Son et al./2021
Dental prostheses	3D DLP digital printing	Photopolymers	-	-	-	[92]/Moraru et al./2018
Removable dental prostheses	Direct light processing (DLP) 3D printer (NextDent 5100)	Dimethacrylate-based	-	-	-	[68]/Jain et al./2021
Dental pros-theses	3D printing machine (STM 125)	Acrylic resin	60	-	-	[93]/Galeva et al.,/2021
Dental crowns	Digital light processing printer (Prodent Labx, Product Bonyan Mecatronic, Tabriz, Iran)	UV resin (Freeprint Temp UV, Detax, Germany)	91.40	-	-	[94]/Mohajeri et al./2021
Dental crowns	DLP-based 3D printer (Hunter, Flashforge Corp., Jinhua, China)	A5AN-500, Nissin Dental Products Inc., Kyoto, Japan			Because of different fabrication angles, there is more than one mean marginal/internal fit	[95]/Ryu et al./2020
Dental crowns	DLP-type 3D printer (NextDent 5100, NextDent, Soesterberg, Netherlands)	PMMA resin liquid (NextDent C&B, NextDent, Soesterberg, Netherlands)	-	-	External surface; mean Trueness; 87.8 μm Intaglio surface; mean Trueness; 78.2 μm	[96]/Lee et al./2021
Dental crowns	Dental SLA 3D printer (ZENITH U; zenith	ZMD-1000B Temporary; Dentis	-	-	-	[97]/Yu et al./2021

3D: three-dimensional; RSB: rounded shoulder with bevel finish line design; KE: knife-edge finish line design; σ_0_: characteristic strength; KI_C_: mean fracture toughness.

## Data Availability

Not applicable.
